# Bone Effects of Binge Alcohol Drinking Using Prepubescent Pigs as a Model

**DOI:** 10.1111/acer.13874

**Published:** 2018-09-12

**Authors:** Ursula Föger‐Samwald, Christian Knecht, Thomas Stimpfl, Thomas Szekeres, Katharina Kerschan‐Schindl, Peter Mikosch, Peter Pietschmann, Wolfgang Sipos

**Affiliations:** ^1^ Department of Pathophysiology and Allergy Research Center for Pathophysiology, Infectiology and Immunology Medical University of Vienna Vienna Austria; ^2^ Clinical Department for Farm Animals and Herd Management University of Veterinary Medicine Vienna Vienna Austria; ^3^ Department of Laboratory Medicine Medical University of Vienna Vienna Austria; ^4^ Department of Physical Medicine and Rehabilitation Medical University of Vienna Vienna Austria; ^5^ Landesklinikum Mistelbach‐Gänserndorf Mistelbach Austria

**Keywords:** Binge Alcohol Drinking, Pigs, Bone Microarchitecture, Calcium, Phosphorus

## Abstract

**Background:**

Although chronic alcohol consumption in adults is an established risk factor for osteoporotic fractures, there is a huge gap in our knowledge about bone effects of binge drinking in adolescents. The aim of this pilot study was therefore to assess skeletal effects of binge alcohol drinking using prepubescent pigs as a large animal model.

**Methods:**

Piglets aged 2 months were offered alcohol orally as a mixture of hard liquor and apple juice. Those with the highest propensity to drink alcohol were included in the experiment and received 1.4 g alcohol/kg bodyweight 2 times per week for 2 months (alcohol group); control piglets received apple juice in an identical manner. At the age of 4 months, the animals were euthanized; trabecular and cortical bone samples from the femur, the tibia, the humerus, and the fourth vertebral body harvested during necropsy were assessed by microcomputed tomography and dynamic histomorphometry. In addition, blood chemistry and blood alcohol determinations were performed.

**Results:**

Blood alcohol levels assessed 1 hour after alcohol administration were 0.99‰ ± 0.15, 1.12‰ ± 0.2, and 1.14‰ ± 0.18 at the ages of 2, 3, and 4 months, respectively. In the alcohol group, serum calcium and phosphate levels were decreased. In the femur, trabecular number and connectivity density were lower in the alcohol than in the control group, and in the humerus and the fourth vertebral bodies, an opposite pattern was seen for trabecular number and connectivity density, respectively. Cortical density was higher in the humerus and trabecular density higher in the tibia of the alcohol group compared to the control group. Cortical porosity was lower in the humerus of the alcohol group. No significant differences were seen for trabecular thickness, trabecular separation, bone volume fraction, and static and dynamic histomorphometric parameters.

**Conclusions:**

In this pilot study, we have assessed skeletal effects of binge alcohol drinking by using prepubescent pigs as a promising large animal model. Binge drinking has bone effects that are site‐specific. However, these data have to be verified in a larger study population.

Alcohol consumption has detrimental effects on various organ systems including also the skeleton. Chronic heavy alcohol consumption is associated with decreased bone mineral density (BMD) and an increased risk of low trauma fractures (Maurel et al., 2012; Mikosch, 2014). In men, chronic alcohol abuse is among the most frequent causes of secondary osteoporosis (Walsh and Eastell, 2013). The pathophysiology of alcohol‐induced osteoporosis is multifactorial. There are indirect effects associated with comorbidities such as malnutrition, vitamin D deficiency, falls, hypogonadism, liver cirrhosis, and lifestyle factors typically associated with alcoholism (e.g., smoking and physical inactivity; Walsh and Eastell, 2013). Direct effects of chronic heavy alcohol consumption have very consistently been demonstrated to target osteoblasts, thereby decreasing bone formation (Luo et al., 2017; Maurel et al., 2012). The effects on osteoclasts, in contrast, are less consistently reported and are described as leading to increased, unchanged, or even decreased activity (Luo et al., 2017; Maurel et al., 2012).

Besides chronic alcohol consumption, in recent years, another pattern of alcohol consumption named binge drinking is increasingly gaining attention as an alarming health problem. The National Institute on Alcohol Abuse and Alcoholism (NIAAA) defines binge drinking as a “pattern of drinking alcohol that brings blood alcohol concentrations to 0.08 gram percent or above,” which “corresponds to the consumption of five standard drinks or more for men and four standard drinks or more for women” (NIAAA, 2004, p. 3). Although not a new phenomenon in adults, a steady rise in binge drinking has been observed especially among female teenagers over the last years (Dwyer‐Lindgren et al., 2015; White and Hingson, 2013). The prevalence in adolescents has been reported to range from 11.2 to 56.9% and to be more frequent in boys and in the upper social classes (Degenhardt et al., 2013; Diehl et al., 2014; Locatelli et al., 2012; Pitel et al., 2011). Studies addressing the effects of binge drinking on the skeleton in adolescent rats report short‐ as well long‐term negative effects on BMD (Lauing et al., 2008), but also increased bone length, bone weight, and bone density (Sampson et al., 1999). Different outcomes in these studies might be due to the different routes of alcohol delivery. Iwaniec and Turner (2013) compared intraperitoneal delivery and oral gavage of alcohol in a rat model of binge drinking. Depending on the delivery route, different effects on bone metabolism were observed. A recent study by LaBrie and colleagues (2018) addressed the effects of binge drinking on the skeleton in adolescent and young adult women. They give evidence for frequent heavy episodic drinking during high school and college years to be associated with decreased vertebral BMD at the ages of 18 to 20 years, whereas reported initiation of heavy episodic drinking before or at the age of 15 years was not significantly related to BMD. Teenagers showing frequent binge drinking behavior might therefore be at an elevated risk of gaining decreased peak bone masses and, as a consequence, suffering from bone disorders such as osteoporosis at advanced ages. Sampson and colleagues (1996) have shown that chronic alcohol consumption in young growing rats reduces bone density and peak bone mass.

Although various studies addressed the effects of alcohol consumption on bone, effects especially of binge drinking on the human skeleton are still insufficiently defined. Human studies struggle with difficulties in the interpretation of data due to confounders and alcoholism‐related comorbidity factors among patients. Moreover, lifetime ethanol (EtOH) consumption is hard to capture. However, especially long‐term alcohol intervention studies are difficult to perform in humans (for review, see Maurel et al., 2012). Except for a few studies in humans assessing the effects of alcohol consumption on markers of bone turnover (Mahabir et al., 2014; Marrone et al., 2012; Nielsen et al., 1990; Sripanyakorn et al., 2009), intervention studies have been mainly performed in experimental animal models with rats being the most widely used animals (Callaci et al., 2004, 2006, 2009; Gaddini et al., 2016; Iwaniec and Turner, 2013; Lauing et al., 2008; Sampson et al., 1999; Wezeman et al., 2007). They offer the advantage of being easy to handle and of short experimental periods. Additionally, these models allow for the analysis of microarchitecture and bone histomorphometry—parameters quite difficult to assess in humans. Nevertheless, the shortcomings of these models also need to be considered. Despite many similarities between rat and human bones, there are also fundamental differences such as bone size, relative amounts of trabecular versus cortical bone, or the absence of intracortical bone remodeling (Lelovas et al., 2008). Nonhuman primates would of course be a suitable animal model due to their very close phylogenetic relationship to humans. However, they are very expensive, difficult to handle, and, at least in small nonhuman primates, notable differences in bone size have to be considered besides ethical concerns. There is no doubt that research on alcohol effects on bone will benefit from alternative, for example, large animal models. Because of several anatomic and physiologic similarities of porcine organ systems and their human orthologs (e.g., bone size, Haversian remodeling, sequence homology of RANKL, monogastric digestive system, omnivore, and aseasonal sexual cycle), the pig is an interesting model for bone research (Sipos et al., 2005, 2011aa). The aim of this study was therefore to investigate the consequences of binge alcohol ingestion on the metabolism and structure of bone using prepubescent pigs as a large animal model.

## Materials and Methods

### Animals and Experimental Setup

Ten male Large White pigs (University Pig Farm, Berndorf, Lower Austria) aged 2 months were randomly assigned to binge alcohol drinking (5 animals) or to the control group (5 animals). Binge alcohol drinking for each piglet consisted of the oral uptake of 1.4 g alcohol/kg bodyweight as a mixture of hard liquor (40% alcohol predilution concentration) and apple juice 2 times per week. Days of alcohol consumption were interrupted by alternate intervals of 2 or 3 days of abstinence. The amount of alcohol for each binge drinking occasion was chosen to correspond to 7 standard drinks (1 standard drink contains 14 g of alcohol; i.e., 98 g alcohol in total) in a man weighing 70 kg and therefore clearly fulfilled the definition of binge drinking (NIAAA, 2004). Piglets randomized to the control group received apple juice and water offered in an otherwise identical manner as in the alcohol group. The mixture of alcohol and apple juice or apple juice alone was given approximately 1 to 2 hours after food intake. Venous blood for the determination of blood chemical markers was drawn at termination of the experiment at the age of 4 months by puncturing the jugular vein. Venous blood for the determination of blood alcohol concentrations was drawn before and 1 hour after alcohol or placebo administration at the ages of 2 (baseline), 3, and 4 months.

As only 2 animals consumed significant amounts of alcohol, an additional experiment with 3 pigs assigned to binge alcohol drinking was performed in a second run. Eight pigs aged 2 months were monitored for their drinking behavior for approximately 1 week. Three of the 8 animals showing the highest propensity to drink alcohol were selected to participate in the additional experiment. The study protocol was the same as for the initial experiment.

All animals were euthanized at the end of the experiment by intracardiac application of T61^®^ (MSD Tiergesundheit, Munich, Germany) after a preceding anesthesia by a ketamine‐azaperone intramuscular (IM) bolus (Sipos et al., 2007). The fourth vertebral body, the humerus, the femur, the tibia, as well as heart and liver samples were collected and preserved in 4% formaldehyde for 3 weeks (bone samples) and 24 hours (heart and liver tissue). Then, samples were stored in 70% EtOH until analysis.

**Figure 1:**
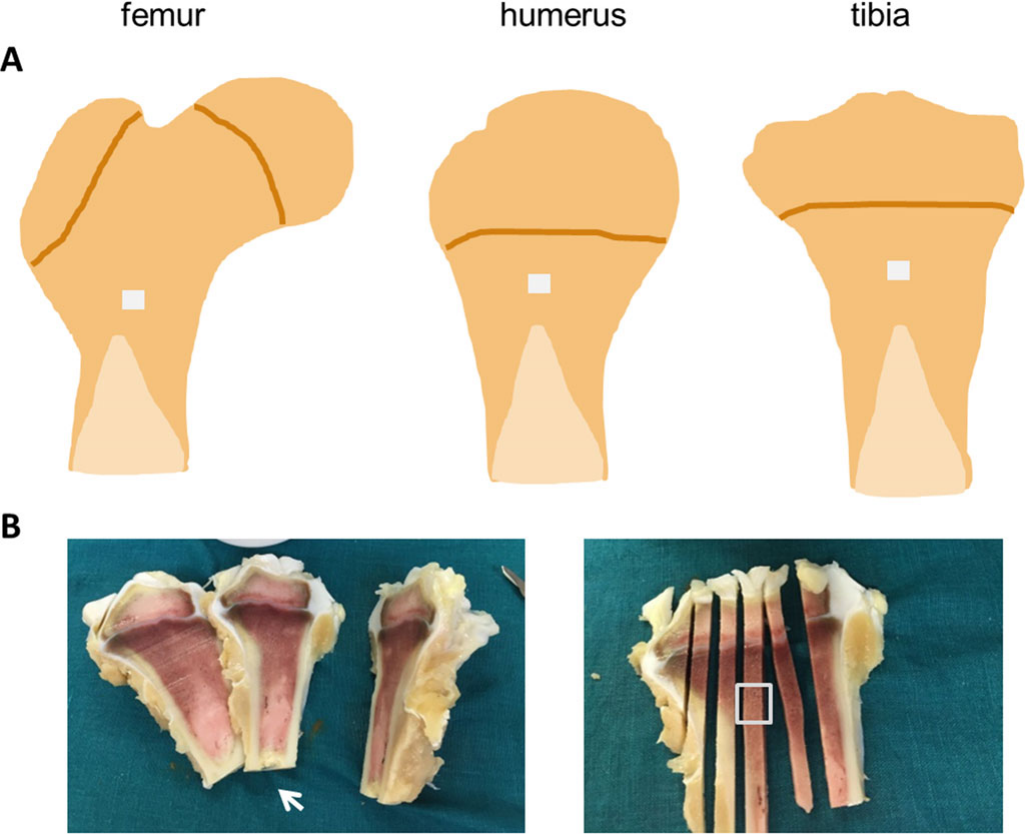
(**A**) Regions in the proximal metaphysis of the femur, humerus, and tibia (**gray cubes**) from where bone samples (approx. 5 × 5 × 5 mm) for histomorphometric and μCT analysis were obtained. (**B**) Representative pictures of bone sampling in the tibia. From a slice (approx. 5 mm thick) (**arrow**) obtained from the central part of the proximal tibia, rods were cut into longitudinal direction. From a rod in the central part of the slice, a cube was cut out for analysis (**rectangle**).

**Figure 2:**
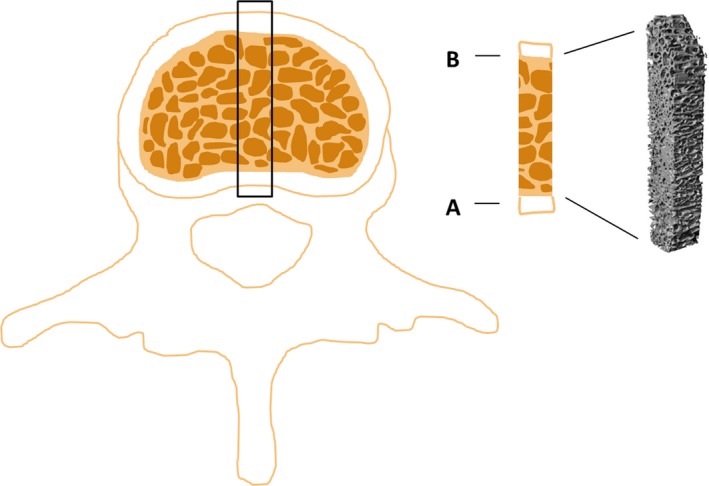
Trabecular bone sample obtained from the fourth vertebral body. The entire region reaching from the dorsal (**A**) to the ventral cortex (**B**) was analyzed by μCT.

**Figure 3:**
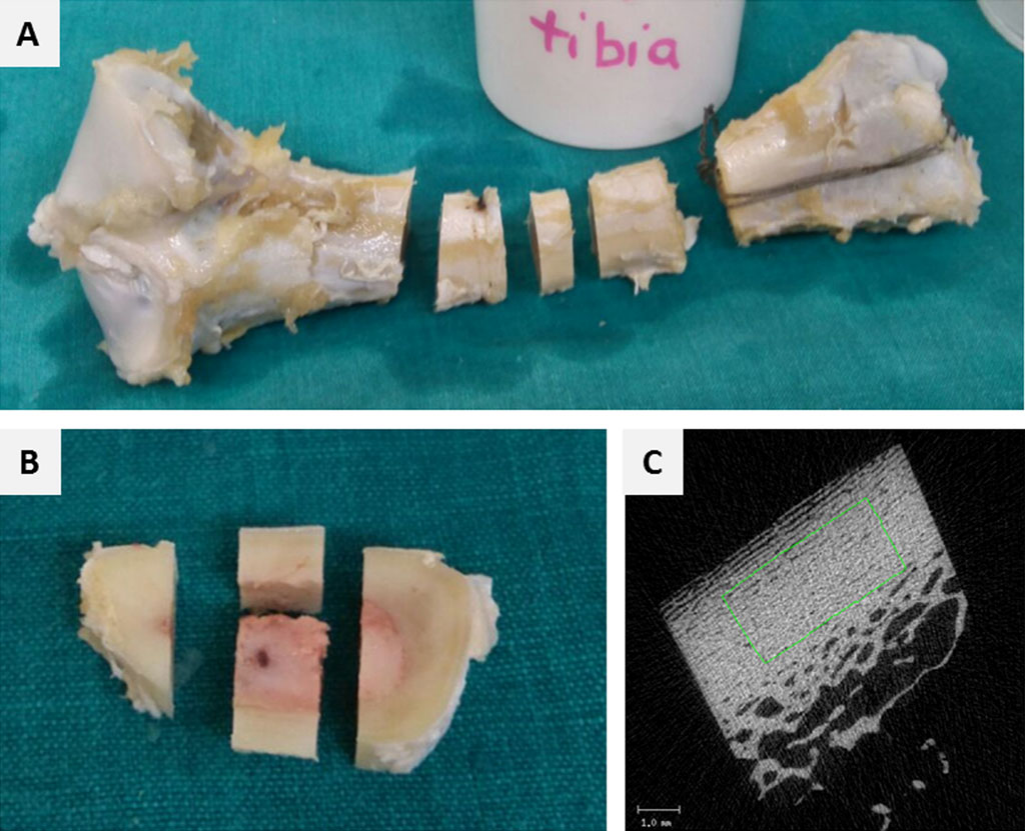
(**A**–**B**) Representative pictures for sampling sites of cortical bone at the midshaft of the tibia. (**A**) A slice approximately 5 mm thick was cut out of the midshaft of long bones with a bone saw. (**B**) From this slice, a cube of approximately 5 × 5 × 5 mm was cut out and (**C**) a cube‐shaped volume of interest (approx. 9 mm^3^) was analyzed by μCT (**C**).

Bone samples for histomorphometric and microcomputed tomography (μCT) analysis were cut out from long bones (femur, tibia, humerus) and the fourth vertebral body with a bone saw (Diamond band saw; Exact, Hamburg, Germany). The region best suited for the analysis of trabecular histomorphometric parameters in long bones was determined by systematically analyzing different regions in the proximal metaphysis and epiphysis of the tibia and the proximal metaphysis and femoral head of the femur. Finally, the analysis was done in a cube‐shaped sample (approx. 5 × 5 × 5 mm) from the central part of the proximal metaphysis of long bones in a region equidistant from the growth plate and the transition from trabecular bone to the bone marrow cavity (tibia, humerus) or a region approximately 5 mm proximal from the transition from trabecular bone to the bone marrow cavity (femur) (Fig. 1). The region best suited for analysis of trabecular histomorphometric parameters in the fourth vertebral body was done by systematically analyzing different regions from the dorsal to the ventral cortex. Finally, analysis was done in a rectangular‐shaped sample (approx. 5 × 5 mm) reaching from the dorsal to the ventral cortex in the central part of the fourth vertebral body (Fig. 2). Bone samples for μCT analysis of trabecular parameters were also taken from the region best suited for analysis of histomorphometric parameters. Cortical bone was analyzed by μCT in a cube (approx. 5 × 5 × 5 mm) cut out from the midshaft of long bones (Fig. 3) with a bone saw (Diamond band saw). A cube‐shaped volume of interest (approx. 4.5 × 2 × 1 mm) in the center of the cortical bone sample was analyzed (Fig. 3).

### Blood Chemistry and Blood Alcohol Titer

To assess the effects of alcohol consumption on blood alcohol levels, mineral metabolism, and liver and kidney function, blood chemical parameters including serum calcium, magnesium, phosphate, activities of glutamate‐pyruvate‐transaminase (GPT), glutamate‐oxaloacetate‐transaminase (GOT), and gamma‐glutamyl transferase (γGT), as well as bilirubin, blood‐urea‐nitrogen (BUN), and blood alcohol levels (Cobas c Analyzer, Roche/Hitachi, Switzerland; detection limit: 10.1 mg/dl; intra‐assay coefficient of variation: 0.4 to 0.8%, interassay coefficient of variation: 1.2 to 2.4%) were analyzed at the Department of Laboratory Medicine, Medical University of Vienna.

### Histomorphometry and μCT

For dynamic histomorphometry, animals were subjected to double tetracycline labeling as described by de Vernejoul and colleagues (1984). Therefore, 20 mg/kg bodyweight oxytetracycline was given IM 2 times at an interval of 7 days and with the second application being performed 2 days before euthanasia. From long bones and the fourth vertebral body collected at necropsy, bone samples approximately 5 × 5 × 5 mm in size were cut out with a bone saw and embedded in methyl methacrylate (K‐Plast; DiaTec, Hallstadt, Germany). Sections of 5 μm were prepared with a rotary microtome (HM 355S; Thermo Scientific, Waltham, MA). Static and dynamic bone histomorphometry was performed in an area of 2.4 mm^2^ in 1 Goldner‐stained and 1 Goldner‐unstained section, per animal, respectively, using an OsteoMeasure system (OsteoMetrics, Decatur, GA). The standardized nomenclature of the ASBMR Histomorphometry Nomenclature Committee was applied (Dempster et al., 2013). Static and dynamic parameters assessed were osteoid volume/bone volume (OV/BV), osteoid surface/bone surface (OS/BS), osteoblast surface/bone surface (Ob.S/BS), number of osteoblasts/bone surface (N.Ob/BS), osteoclast surface/bone surface (Oc.S/BS), number of osteoclasts/bone surface (N.Oc/BS), mineralized surface/bone surface (MS/BS) calculated as double‐labeled plus 50% single‐labeled surface per bone surface, mineral apposition rate (MAR), and bone formation rate/bone surface (BFR/BS).

Cortical and trabecular samples of the fourth vertebral body, the humerus, the femur, and the tibia cut out with a bone saw were analyzed by μCT (MicroCT 35, Scanco, Switzerland). The X‐ray tube was operated at 70 kVp and 114 μA with an integration time set to 300 ms. Scans were performed at an isotropic, nominal, and spatial resolution of 10 μm (high‐resolution mode). A threshold of 220 and 260 was used in the trabecular and cortical compartment, respectively, to separate bone from nonbone tissue. Trabecular parameters assessed were connectivity density (Conn.D.), bone volume/total volume (BV/TV), trabecular number (Tb.N), trabecular thickness (Tb.Th), trabecular separation (Tb.Sp), and trabecular bone mineral density (Tb.BMD). Cortical parameters assessed were cortical porosity, mean pore diameter, and cortical bone mineral density (Ct.BMD). Cortical porosity expressed as percent was calculated by multiplying the difference of 1 and BV/TV by 100.

### Statistics

Statistical differences between the alcohol and the control group were assessed with Mann‐Whitney *U*‐test.

### Legal and Ethical Considerations

This experiment was performed according to Austrian Law and Animal Ethics Guidelines (animal experiment number: GZ 68.205/0061‐WF/V/3b/2015).

## Results

### Blood Chemistry and Histology

Alcohol offered was consumed over an interval of approximately 10 minutes. In some cases, not the whole amount of alcohol offered was consumed. Moreover, control of the exact volume of voluntarily consumed fluids in pigs is nearly impossible, as you cannot prevent pigs from “playing” with fluids or feed. For these reasons, the essential criterion for inclusion into the alcohol group was not the amount of alcohol consumed but blood alcohol levels measured 1 hour after alcohol intake. Baseline blood alcohol levels were below the detection limit in all animals. In only 2 of 5 animals of the initial alcohol group, blood alcohol levels above 0.8‰ were measured 1 hour after alcohol intake at all 3 time points (2, 3, and 4 months). In 1 animal, blood alcohol levels were below the detection limit 1 hour after alcohol intake for all time points measured. These data correspond to our observation that the propensity to drink alcohol differed between the 5 animals of this group. Taken together, from the 5 animals of the alcohol group of the initial experiment, only 2 animals consistently consumed alcohol. For this reason, a second experiment with a minor modification was done. Three additional animals, whose propensity for alcohol by daily offerings of small amounts of alcohol was assessed by an observation period of 1 week prior to the start of the experiment, were recruited for the alcohol group. All 3 animals had blood alcohol levels above 0.8‰ at the ages of 2, 3, and 4 months. Thus, for further analysis, 5 animals in total with a mean alcohol concentration of 0.99‰ ± 0.15, 1.12‰ ± 0.2, and 1.14‰ ± 0.18 1 hour after alcohol intake at the ages of 2, 3, and 4 months, respectively, were available (Table 1).

**Table 1:** Blood Alcohol Concentrations (‰) Assessed in the 5 Animals of the Alcohol Group (#1 to #5) 1 Hour After Alcohol Administration at the Ages of 2, 3, and 4 Months

Animal	2 months	3 months	4 months
#1	0.80	1.20	1.08
#2	0.98	0.88	1.17
#3	1.00	1.00	1.42
#4	1.22	1.13	1.10
#5	0.95	1.40	0.94
Means ± SD	0.99 ± 0.15	1.12 ± 0.2	1.14 ± 0.18

SD, standard deviation.

Blood chemistry parameters are given in Table 2. Serum calcium and phosphate levels were significantly lower in the alcohol compared to the control group. No significant differences were seen for all other parameters assessed. In heart and liver histology, no abnormalities were seen.

**Table 2:** Blood Chemistry Parameters Assessed at the Age of 4 Months

	Control median	Control min; max	Alcohol median	Alcohol min; max	*p*
GPT (U/l)	61.0	43.0;85.0	73.0	58.0;97.0	0.55
GOT (U/l)	35.0	30.0;126.0	53.0	37.0;220.0	0.10
γGT (U/l)	39.0	27.0;58.0	39.0	23.0;486.0	1.00
Bilirubin (mg/dl)	0.02	0.01;0.03	0.03	0.01;0.03	0.57
BUN (mg/dl)	12.2	8.7;15.1	12.2	9.7;15.5	0.75
Ca (mmol/l)	2.71	2.55;2.76	2.25	1.20;2.67	0.05*
Mg (mmol/l)	0.90	0.83;0.98	0.82	0.03;0.93	0.10
P (mmol/l)	3.01	2.70;3.13	2.37	2.11;2.58	0.01**

Statistical differences between the 2 groups were determined with Mann‐Whitney *U*‐test (*p ≤ 0.05, **p ≤ 0.01).

### Behavioral Observations

Approximately 15 minutes after alcohol intake, pigs showed an uncoordinated gait, leaned against the walls, became drowsy, and finally laid down. Then, they started to snore and fell asleep. The sleeping phase lasted between 1 and several hours. When they woke up again, pigs did not show any signs of hangover such as behavioral depression, isolation from the group, or refusal to take up food.

### Bone Samples for Trabecular Histomorphometry and μCT Analysis

#### Long Bones

**Figure 4:**
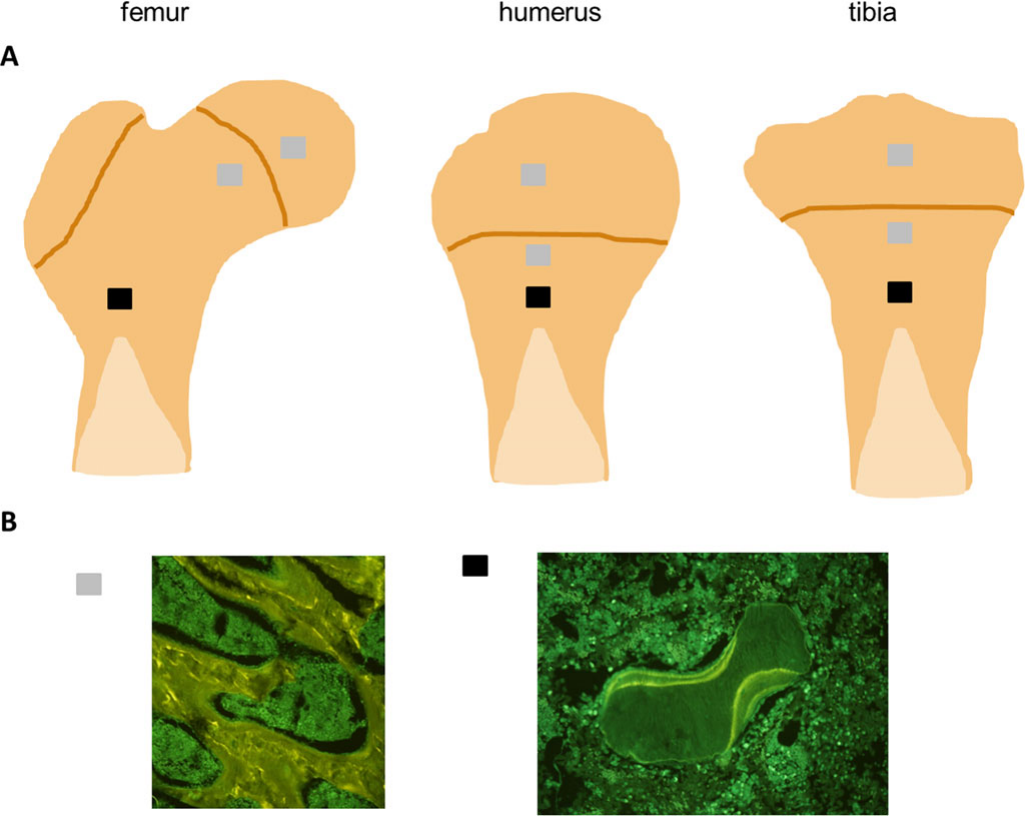
(**A**) Samples (approx. 5 × 5 × 5 mm) for histomorphometric analysis obtained from the head and metaphysis of the femur, and the epiphysis and metaphysis of the humerus, and the tibia. (**B**) Representative images of tetracycline labels in samples obtained from more proximal regions (**gray boxes**) and from more distal regions (**black boxes**).

In a systematical assessment of different regions of the proximal femur, tibia, and humerus by histomorphometry, we found blurred tetracycline stains (probably indicating the presence of primary spongiosa) in the femoral head and the epiphysis of the humerus and the tibia (Fig. 4). The analysis of dynamic histomorphometric parameters was therefore not reasonable in these regions. Finally, we identified a region equidistant from the growth plate and the transition from trabecular bone to the bone marrow cavity in the proximal metaphysis of the tibia and the humerus and a region approximately 5 mm proximal from the transition from trabecular bone to the bone marrow cavity in the proximal metaphysis of the femur suitable for analysis of histomorphometric and μCT parameters (Fig. 1).

#### Vertebrae

By doing a systematical assessment of different regions of the vertebral body, we found no blurred tetracycline stains in any of the regions analyzed. We chose a cube (approx. 5 × 5 × 5 mm) from the trabecular central part of the fourth vertebral body for the analysis of histomorphometric parameters. The entire trabecular region reaching from the dorsal to the ventral cortex was analyzed by μCT in a rectangular (approx. 5 × 5 mm) sample obtained from the central part of the fourth vertebral body (Fig. 2).

### Microcomputed Tomography

#### Trabecular Bone

**Figure 5:**
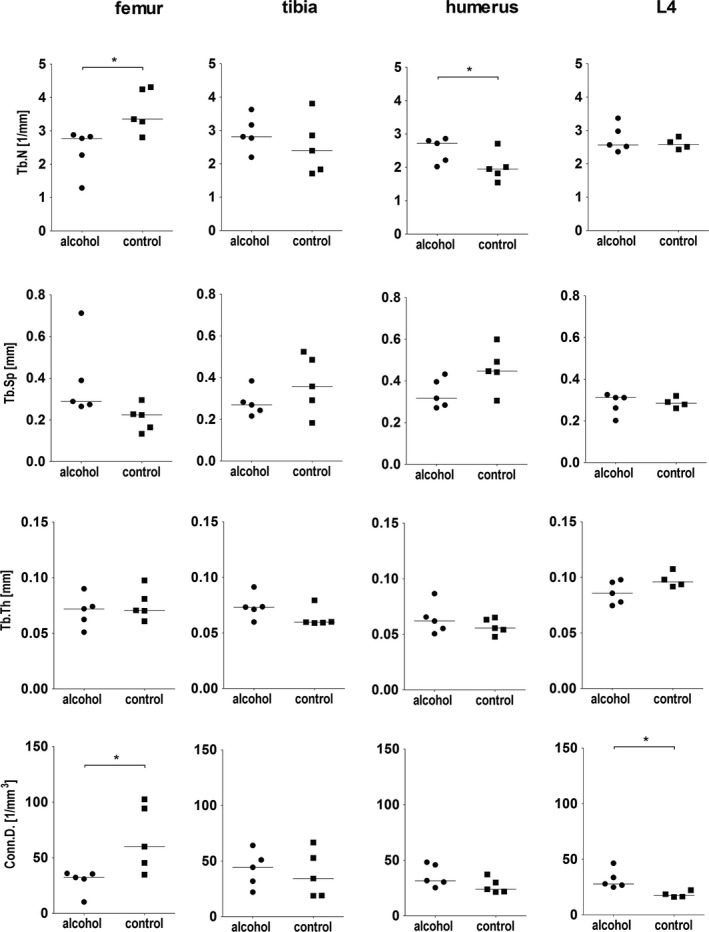
Comparison of trabecular parameters in the femur, the tibia, the humerus, and the fourth vertebral body (L4) between the alcohol (*n* = 5) and the control group (*n* = 5, for L4 *n* = 4). Statistical differences between the 2 groups were assessed with Mann‐Whitney *U*‐test (*p ≤ 0.05).

**Figure 6:**
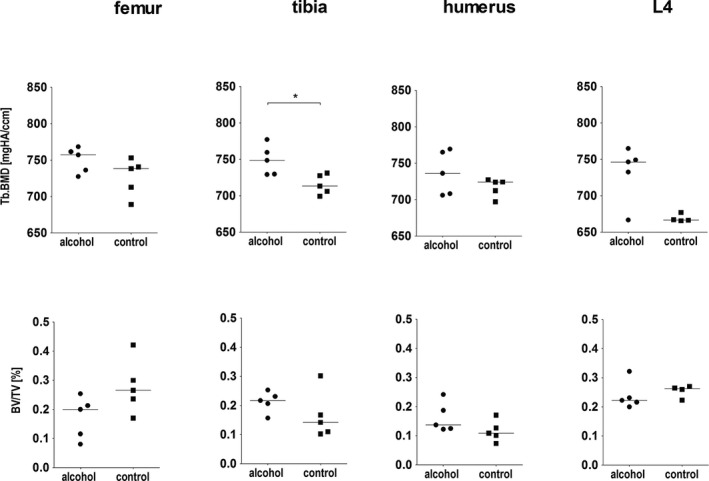
Comparison of trabecular parameters in the femur, the tibia, the humerus, and the fourth vertebral body (L4) between the alcohol (*n* = 5) and the control group (*n* = 5, for L4 *n* = 4). Statistical differences between the 2 groups were assessed with Mann‐Whitney *U*‐test (*p ≤ 0.05).

A comparison of trabecular parameters between the alcohol and the control group is given in Figs 5 and 6. In the femur, Conn.D. was significantly lower, whereas in the vertebrae, Conn.D. was significantly higher in the alcohol group. In the femur, statistical differences were seen also for Tb.N, which was significantly lower in the alcohol group. In the humerus, an opposite pattern with a significantly higher Tb.N in the alcohol group was seen. Tb.BMD was significantly higher in the alcohol group in the tibia. No significant differences were seen for the other parameters assessed.

#### Cortical Bone

**Figure 7:**
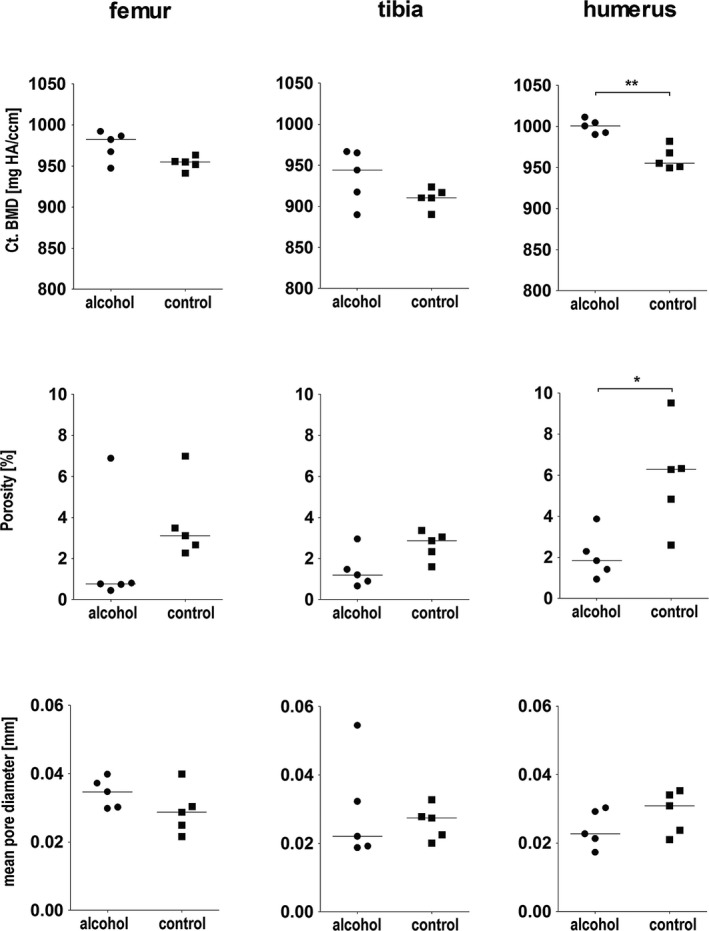
Comparison of cortical parameters in the femur, the tibia, and the humerus between the alcohol group (*n* = 5) and the control group (*n* = 5). Statistical differences between the 2 groups were assessed with Mann‐Whitney *U*‐test (*p ≤ 0.05, **p ≤ 0.01).

A comparison of cortical parameters in the femur, the tibia, and the humerus between the alcohol and the control group is given in Fig. 7. In the humerus, cortical porosity was significantly lower and cortical density was significantly higher in the alcohol group. In the femur and the tibia, no significant differences but a trend toward lower cortical porosity (*p* ≤ 0.1) and in the femur but not in the tibia a trend toward higher cortical density (*p* ≤ 0.1) were observed in the alcohol group. No significant differences were seen for the mean pore diameter.

### Static and Dynamic Histomorphometry

**Figure 8:**
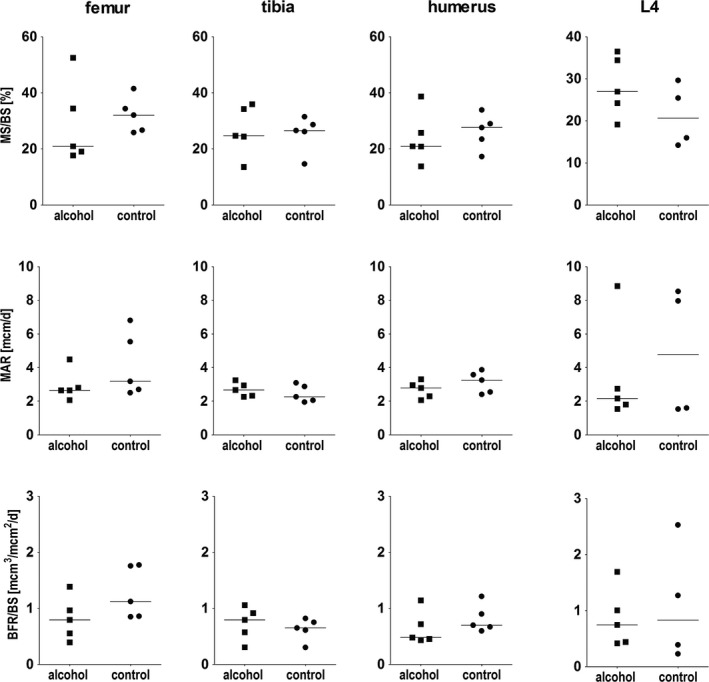
Comparison of dynamic histomorphometric parameters in the femur, the humerus, the tibia, and the fourth vertebral body (L4) between the alcohol group (*n* = 5) and the control group (*n* = 5, for L4 *n* = 4).

**Figure 9:**
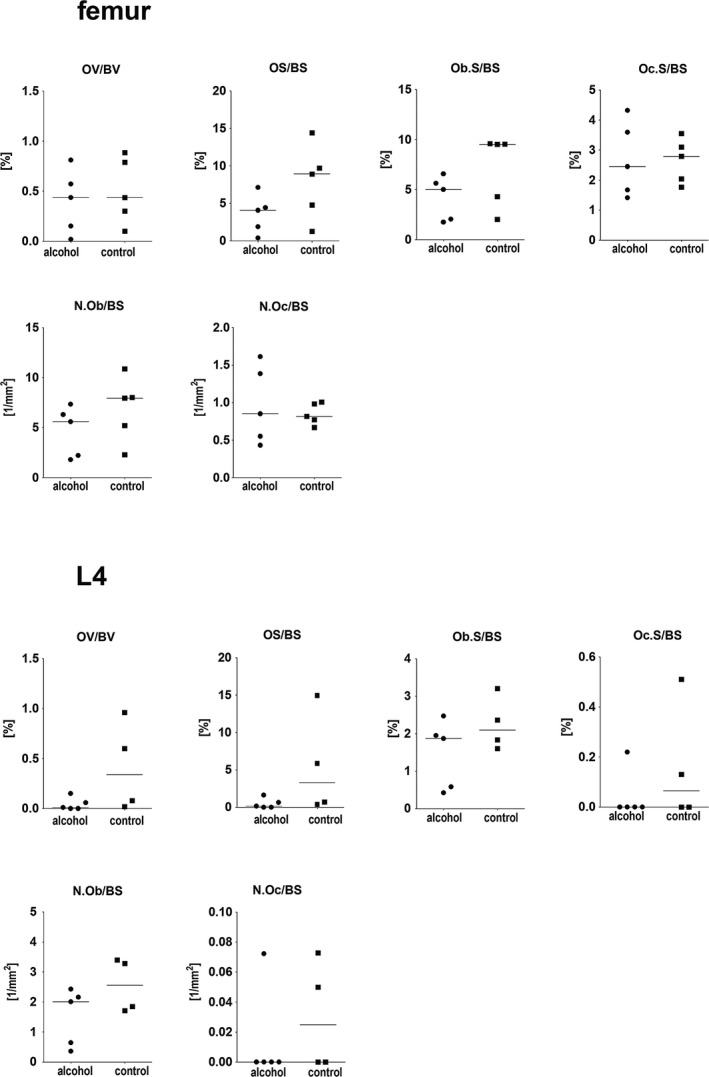
Comparison of static histomorphometric parameters in the femur and the fourth vertebral body (L4) between the alcohol group (*n* = 5) and the control group (*n* = 5, for L4 *n* = 4).

No significant differences between the 2 groups were observed neither for static nor for dynamic histomorphometric parameters (Figs 8 and 9).

## Discussion

We used prepubescent pigs as a large animal model to assess skeletal effects of binge drinking. To our knowledge, this is the first study to examine the effects of binge drinking on the skeletal system in pigs.

When choosing a specific species as animal model in medical research, anatomical and physiological analogies in comparison with humans are fundamental requirements that have to be considered. Swine are a widely used animal species as surgical models, in translational research and in preclinical toxicological testing of pharmaceuticals. This is mainly due to major similarities concerning the cardiovascular, urinary, integumentary, and digestive systems (for review, see Swindle et al., 2012). Major similarities have also been found with respect to bone biology, making them suitable models for a variety of bone‐related studies including studies of bone fractures, of bone ingrowth, or implant biomaterial research (for review, see Pearce et al., 2007). Swine have been found to show a high comparability with humans concerning bone micro‐ and macrostructure (Pearce et al., 2007). When using swine as a model for studying effects of alcohol drinking on bone, especially similarities concerning bone physiology are of crucial importance. Our group showed that porcine bone marrow stromal cells express a cytokine pattern including the RANK/RANKL/OPG system comparable to human and murine cells (Sipos et al., 2005, 2011bb). Moreover, bone remodeling, the process responsible for maintaining bone integrity and mineral homeostasis, has been described to be similar to humans. Swine, in contrast to rodents, show trabecular as well as osteonal bone remodeling (Hillier and Bell, 2007; Lelovas et al., 2008). They are therefore suitable models for studying the effects of alcohol on trabecular as well as cortical bone structure and physiology.

The method of alcohol administration is crucial to ensure comparability to human alcohol consumption. A technique used in several studies is intraperitoneal injection of alcohol. This is very efficient to achieve high blood alcohol concentrations, but is stressful for the animals. Moreover, alcohol absorption by peripheral tissue may occur. Alcohol uptake by the body may therefore differ from humans (for review, see Maurel et al., 2012). In line with this, Iwaniec and Turner (2013) observed the effects of intraperitoneal injection of EtOH on bone metabolism in rats that were not seen when EtOH was administered by oral gavage. Oral supply as a liquid is the technique of alcohol administration, which most closely resembles human drinking behavior and which was applied in this study. This technique presupposes voluntary consumption of large quantities of alcohol as it is seen in humans. Swine are known to voluntarily consume alcohol to the point of intoxication (Cudd, 2008). This is what we also observed in our study. However, the propensity to alcohol drinking differed between the study animals with some of them refraining from alcohol drinking over the whole or over part of the experimental period. We therefore established a study protocol including a premonitory step, in which pigs were monitored for their drinking behavior. Only those with a high propensity to alcohol drinking were included in the experiment. Eventually, animals included in the alcohol group had blood alcohol levels above 0.8‰ at the ages of 2, 3, and 4 months 1 hour after alcohol intake. The dose of alcohol offered (1.4 g alcohol/kg bodyweight) and the time schedule of alcohol administration were therefore confirmed to be sufficient to mimic binge alcohol drinking in humans as defined by the NIAAA (2004). Lower phosphate and calcium serum levels as observed in the alcohol compared to the control group are also associated with alcohol abuse in humans (Mikosch, 2014; Peterlik, 2007) and thereby further support the comparability of our model system to the effects of alcohol abuse in humans.

The effects of binge drinking on the skeletal system were analyzed by various methods, among them structural and histomorphometric analysis of bone samples were from different sites. Careful selection of the regions within the different sites from which the samples were obtained was a crucial step in our study. We did a thorough screening of different regions and found a large heterogeneity within the different sites with regard to the pattern of fluorescence labeling especially in the proximal regions of the femur, the tibia, and the humerus. Proximal from the growth plate tetracycline labels were blurred probably indicating the presence of primary spongiosa. As the analysis of dynamic histomorphometric parameters was not reasonable in this region, we identified a region equidistant from the growth plate and the transition from trabecular bone to the bone marrow cavity in the tibia and the humerus and a region approximately 5 mm proximal from the transition from trabecular bone to the bone marrow cavity in the femur suitable for evaluation of dynamic histomorphometric parameters. To allow for comparison, bone samples for μCT analysis were also taken from this region.

The assessment of skeletal effects of binge drinking in this study included the analysis of trabecular and cortical microstructural parameters besides the assessment of BMD. The effects of alcohol on bone quality and bone architecture in humans have mainly been described by changes in BMD, a parameter accessible by noninvasive methods (for review, see Maurel et al., 2012; Mikosch, 2014). However, as bone microarchitecture is an important determinant of bone strength and in consequence of fracture risk (Seeman and Delmas, 2006), analysis of microarchitecture is crucial to provide a comprehensive picture of alcohol impact on bone. There are new noninvasive techniques available to assess bone microarchitecture in humans. However, they are restricted to non–weight‐bearing sites of the distal arms and legs. Animal studies can therefore add valuable insights into the effects of alcohol on bone strength and fracture risk. We analyzed microarchitecture in 4 different bones: the femur, the tibia, the humerus, and the fourth vertebral body. With regard to alcohol effects, substantial differences between these 4 sites were observed. Whereas Tb.N and Conn.D. were lower in the femur of the alcohol group, the opposite pattern concerning Conn.D. and the opposite pattern concerning Tb.N were seen in the fourth vertebral body and the humerus, respectively. Following these data, skeletal heterogeneity, which proposes differences between different sites of the body in essential characteristics of bone including bone structure, mechanical properties, or bone metabolism, can be seen with regard to alcohol effects on bone. The analysis of more than 1 site is therefore important to prevent drawing invalid conclusions from 1 site on a general impact of alcohol on bone.

Studies in rodents, the most widely used experimental animal models for studying the effects of binge alcohol consumption on bone, have mainly focused on cancellous bone and on micro‐ and macrostructural aspects of cortical bone (Callaci et al., 2004, 2006, 2009; Iwaniec and Turner, 2013; Lauing et al., 2008; Sampson et al., 1999; Wezeman et al., 2007). Data on cortical bone metabolism are not available as intracortical (osteonal) bone remodeling is absent in small rodents. However, cortical bone not only constitutes 80% of the skeleton, but also substantially contributes to bone strength. Characteristic fractures seen in the elderly are fractures in typical cortical sites such as the hip due to the increased intracortical and endocortical remodeling (Seeman, 2013). Underlining an impact of alcohol on cortical bone, consumption of more than 2 drinks per day is associated with higher risks to sustain a hip fracture (Kanis et al., 2005). Gaddini and colleagues (2015) investigated the effects of long‐duration (12 months) heavy alcohol consumption on cortical bone remodeling of the tibia in late adolescent male rhesus macaques. They found no differences for bone mineral content, BMD, or cortical bone architecture. Cortical porosity and labeled osteon density were lower in the alcohol group compared to the control group, suggesting a reduced rate of cortical bone remodeling as interpreted by the authors. These data are in line with our observations of lower cortical porosity in the alcohol compared to the control group and thereby suggest similar mechanisms of alcohol action in these 2 nonrodent models (Gaddini et al., 2015). Taken together, with our model, valuable additional insights into the impact of alcohol consumption on cortical bone metabolism can be gained.

Considering all changes observed in this study under the influence of binge alcohol consumption, no consistent effect with respect to the site investigated could be seen. The most consistent effect observed with respect to the parameters investigated was an increase in BMD in the alcohol compared to the control group. This is in contrast to observations of either lower or unchanged BMD values observed in most rat models of binge drinking done so far (Callaci et al., 2004, 2006, 2009; Lauing et al., 2008; Wezeman et al., 2007). Also, in the study of LaBrie and colleagues (2018), frequent binge drinking during high school and college years was associated with decreased vertebral BMD in women aged 18 to 20 years. However, improved indices of bone metabolism and bone architecture were observed in a rat model of binge drinking with oral gavage of alcohol (Sampson et al., 1999). The rat models of binge drinking cited above (Callaci et al., 2004, 2006, 2009; Lauing et al., 2008; Wezeman et al., 2007) applied intraperitoneal delivery of alcohol. Different effects of oral and intraperitoneal delivery of alcohol on bone by direct comparison in a rat model of binge alcohol drinking have also been shown by Iwaniec and Turner (2013). Higher BMD values associated with alcohol consumption in humans were reported in the context of light alcohol consumption, and, depending on sex, age, hormonal status, and type of beverage consumed, higher or lower BMD was reported with moderate alcohol consumption (for review, see Maurel et al., 2012). Bone effects seen with binge alcohol consumption in our study might therefore resemble more the bone effects seen with light than with heavy chronic alcohol consumption. However, as not all animals in this study were littermates and we cannot exclude that the differences observed in this study are exclusively due to alcohol administration, these results have to be verified in further experiments.

A limitation of this study is the small sample size of 5 animals per group. Despite this, several differences in microstructural parameters assessed by μCT were statistically significant. The histomorphometric analysis provided no statistically significant differences. However, data gathered in this study provide valuable information to perform a sample size calculation for follow‐up studies. Another limitation is that a second part of the experiment was performed time‐delayed to the first part. For this reason, animal background may have differed, thus leading to data interpretation difficulties.

In conclusion, we used a novel porcine model to study bone effects of voluntary prepubescent binge alcohol drinking that includes a premonitory step to identify those animals with the highest propensity to alcohol drinking. In contrast to rodent models, pigs also allow for analysis of cortical bone remodeling. Future studies in pigs will add valuable insights into alcohol impact on trabecular and cortical bone strength and might also be used to study complex secondary effects of alcohol on bone by other organ systems, for example, the liver or the immune system. Moreover, pigs might be used to study long‐term effects of binge alcohol drinking at a young age on the bone compartment.

## Funding Source

This work was supported by the Austrian Science Fund (FWF, Grant Nr.: P28827‐B30).

## Conflict of interest

The authors have no conflict of interest to declare.
